# Thyroid Peroxidase Gene Mutation in Patients with Congenital Hypothyroidism in Isfahan, Iran

**DOI:** 10.1155/2012/717283

**Published:** 2012-08-02

**Authors:** Mahin Hashemipour, Fahimeh Soheilipour, Sakineh Karimizare, Hossein Khanahmad, Morteza Karimipour, Sepideh Aminzadeh, Leila Kokabee, Massoud Amini, Silva Hovsepian, Rezvaneh Hadian

**Affiliations:** ^1^Isfahan Endocrine and Metabolism Research Center, Child Growth and Development Research Center, Isfahan University of Medical Sciences, Isfahan, Iran; ^2^Molecular Medicine Department, Pasteur Institute of Iran, Tehran, Iran; ^3^Isfahan Endocrine and Metabolism Research Center, Isfahan University of Medical Sciences, Isfahan, Iran

## Abstract

*Background*. Thyroid peroxidase gene (TPO) mutations are one of the most common causes of thyroid dyshormonogenesis in patients with congenital hypothyroidism (CH). In this study, the prevalence of TPO gene mutations in patients with thyroid dyshormonogenesis in Isfahan was investigated. *Methods*. In this cross-sectional study, genomic DNA of 41 patients with permanent CH due to thyroid dyshormonogenesis was extracted using the salting out method. The 17 exonic regions of the TPO gene were amplified. SSCP technique was performed for scanning of the exonic regions of the TPO gene, except exon 8. DNA sequencing was performed for those with different migration patterns in SSCP by chain termination method. Exon 8 was sequenced directly in all patients. In 4 patients, all fragments were also sequenced. *Results*. One missense mutation c.2669G > A (NM_000547.5) at exon 15 (14th coding exon) in one patient in homozygous form and seven different single nucleotide polymorphisms (SNPs) in exons 1, 7, 8, 11, and 15 of TPO gene. *Conclusion*. The TPO gene mutations among CH patients with dyshormonogenesis in Isfahan were less frequent in comparison with other similar studies. It may be due to the presence of other unknown gene mutations which could not be detected by SSCP and sequencing methods.

## 1. Introduction

Congenital hypothyroidism (CH) is the most common congenital endocrine disorder in childhood which is associated with irreversible neurological problems and poor growth in untreated individuals. In Europe and North America, 1 in 3,000 to 4,000 newborns is affected by this disease [1, 2].

In the majority of patients, CH is sporadic and caused by an abnormal development of the thyroid gland (thyroid dysgenesis). Hereditary inborn errors in the enzymatic cascade of thyroid hormone synthesis are accounted in 20% of all cases which is defined as thyroid dyshormonogenesis. This disorder typically transmitted in an autosomal recessive manner [3].

Of the several genetic defects responsible for thyroid dyshormonogenesis, mutations in thyroid peroxidase (TPO) gene are the most prevalent causes of inherited defects in CH [4]. The TPO gene is located on chromosome 2p25, containing 17 exons encoding a protein of 933 amino acids. Thyroid peroxidase protein is a membrane-bound enzyme which involves in the biosynthesis of thyroid hormones [5]. TPO mutations have been described in various ethnic populations. So far more than 60 inactivating mutations associated with TPO gene have been identified including missense and nonsense mutations, splicing errors, deletions, and insertions of nucleotides. Prevalent mutations are in exons 8, 9, 10, and 11 (catalytic site) [6–24].

Since 2002, the neonatal screening program for CH has been initiated in Isfahan, Iran. The incidence of CH in this population was estimated to be 1 : 357 newborns which is about 10 times higher than reports from North America and Europe [25].

This difference is more likely due to iodine deficiency which is the main cause of transient CH [26]. According to the recent studies, Isfahan population has become iodide sufficient [27]. But the rate of permanent CH in Isfahan is higher than the comparable worldwide rates [28]. Further investigation has shown that thyroid dyshormonogenesis is the most common aetiology of CH in this population [29, 30].

Considering the high prevalence of thyroid dyshormonogenesis in Isfahan and the role of TPO gene mutation in the etiology of this type of CH, in the present study, the frequency of TPO gene defects in patients with thyroid dyshormonogenesis was detected.

## 2. Material and Methods

### 2.1. Patients

In this cross-sectional study, 41 dyshormonogenetic CH patients, diagnosed and followed up during CH screening program in Isfahan Endocrine and Metabolism Research Center, were enrolled. The Medical Ethics Committee of the Isfahan Endocrine and Metabolism Research Center approved the study protocol, and parents of all selected CH patients gave their written consent.

According to CH screening guideline, neonates with screening TSH level of >10 mIU/L at 3–7 days of life recalled. The newborns with abnormal screening results were reexamined on 7th–15th days of birth. Neonates were considered as CH when having TSH > 10 mIU/L and T_4_ < 6.5 mg/dL in second measurements. Thyroid hormone replacement therapy was started in the form of L-T_4_ (levothyroxine) within the first 2 weeks of life. Permanent cases were determined at 3 years old by measuring TSH and T4 concentration 4 weeks after withdrawal of L-T_4_ levothyroxine therapy. Patients with elevated TSH levels (TSH > 10 mIU/L) and decreased T_4_ levels (T_4_ < 6.5 *μ*g/dL) at this time were considered as permanent CH. The etiology of CH was determined by thyroid scan and/or ultrasound before treatment in neonatal period or at age of 3 years old after confirming the permanency of CH. Patients with thyroid gland of normal size according to radiologic findings (i.e., those without thyroid agenesis, hypoplasis, hemiagenesis, or ectopia) were considered to have dyshormonogenesis.

Peripheral blood samples were obtained from selected patients and transferred to Molecular Medicine Department of Pasteur Institute of Iran for molecular analysis and determining the TPO gene mutations.

Serum T_4_ and TSH were measured by radioimmunoassay (RIA) and immunoradiometric assay (IRMA) methods, respectively.

### 2.2. DNA Isolation and Amplification

Genomic DNA was extracted from white peripheral blood cells using the salting out method [31].

Primers were specially designed using the computer program (Gene Runner), for all of the 17 exons and exon-intron boundaries of TPO gene. Their oligonucleotide sequences and the position of their 5′coding sequence ends are listed in Table 1.

The 17 exonic regions of the TPO gene, including the splicing regions, were amplified by polymerase chain reaction (PCR). The PCR reaction mixture contained 10 pmol of each forward and reverse primers, 500 ng genomic DNA, 2 mM MgCl_2_, 200 *μ*M of each dNTP (Cinnagene, Iran), 2.5 *μ*L 10x PCR buffer, 0.5 U Taq DNA polymerase (Cinnagene, Iran) at a final volume of 25 *μ*L. For amplification of exon 8, 1 *μ*L dimethyl sulfoxide (DMSO) was added. The PCR reactions were performed in a thermal cycler machine (Eppendorf, Germany) with an initial denaturation of 10 min at 95°C, followed by 30 cycles of amplification consisting of denaturation at 95°C for 50 second, annealing at 55–62°C (depend on suitable annealing temperature for each primer) for 40 seconds and extension at 72°C for 30–60 seconds (depending on PCR products length) and with a final extension at 72°C for 5 min.

### 2.3. Single Strand Conformation Polymorphism (SSCP) ****Analysis and DNA Sequencing

All amplified PCR products except exon 8 of TPO gene were screened by single-strand conformational polymorphism analysis (SSCP) from 41 selected patients and normal controls. The gel matrix for SSCP contained 8% polyacrylamide gel (29 : 1 or 39 : 1) (Qiagen, Germany) with 3% glycerol. For SSCP, 5 *μ*L of PCR products were first mixed with a 7 *μ*L SSCP loading buffer (xylene cyanol 0·05%, bromophenol blue 0·05%, formamide 95%), the mixture was incubated at 95°C for 10 minutes and then was transferred quickly in to ice bath. Samples were electrophoresed for 10–16 hours at a constant temperature (4°C). Gels were stained by standard silver staining method to visualizing DNA. Fragments presenting different migration pattern in comparison with normal controls were directly sequenced for nucleotide change identification.

Exon 8 of TPO gene was sequenced directly in all patients. In 4 patients, all fragments were sequenced besides SSCP analysis. For sequencing, PCR fragments were purified by DNA Gel Extraction Kit (Qiagen, Germany). Sequencing analysis was done based on chain termination method, using forward and reverse primers in Table 1.

### 2.4. Data Analysis

Sequences were analyzed, using chromas program and compared with the normal TPO gene sequence (Gen Bank Accession number: DQ011222) by BLAST online software (http://www.ncbi.nlm.nih.gov/blast/). Nucleotide changes were compared with mutation database of TPO gene (http://www.hgmd.cf.ac.uk/). 

## 3. Results

 In this study, 41 patients (15 male and 18 female) with dyshormonogenetic congenital hypothyroidism were evaluated for TPO gene mutation. Mean age of studied population was 44.6 ± 5.7 months. Mean of screening TSH and T_4_ level in studied population was 46.2 ± 37.1 (mIU/L) and 6.0 ± 2.8 (*μ*g/dL), respectively. 63% (26/41) of patients had parental consanguinity (18 of them had first-degree parental consanguinity). None of them had goiter during clinical examination.

**Figure 1:**
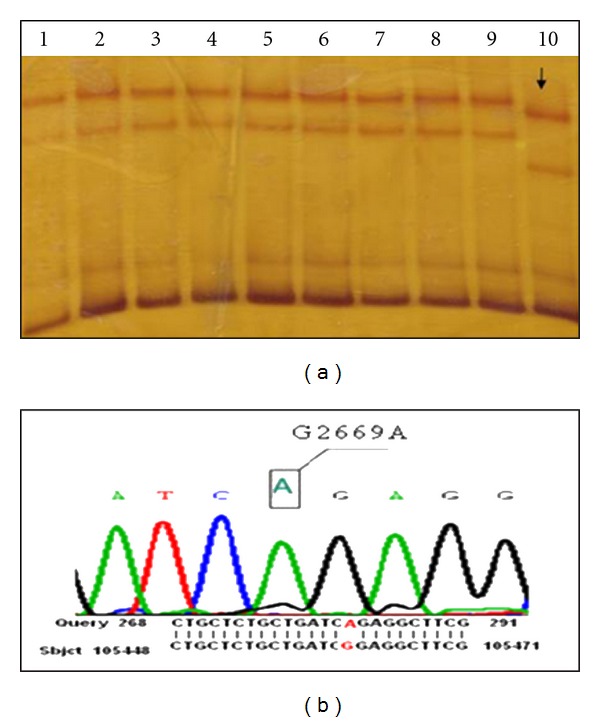
(a) SSCP analysis of exon 15 fragments of thyroid peroxidase (TPO) gene in nine patients with thyroid dyshormonogenesis of Isfahan, Iran. Line 1: normal control. Line 2–8:  patients that did not have aberrant shift and mutation in exon 15. Line 9: the patient with homozygous G2669A mutation in exon 15. (b) Sequencing analysis result of patient with homozygous G2669A mutation in exon 15.

TPO gene mutation was detected only in one patient. The mutation was located in exon 15 (14th coding exon) of TPO gene at nucleotide position c.2669G > A (NM_000547.5) (Figure 1). This mutation results in a glycine to arginine substitution at amino acid position 860 p.Gly860Arg in homozygous form. This mutation has been described previously in the database [17].

**Figure 2:**
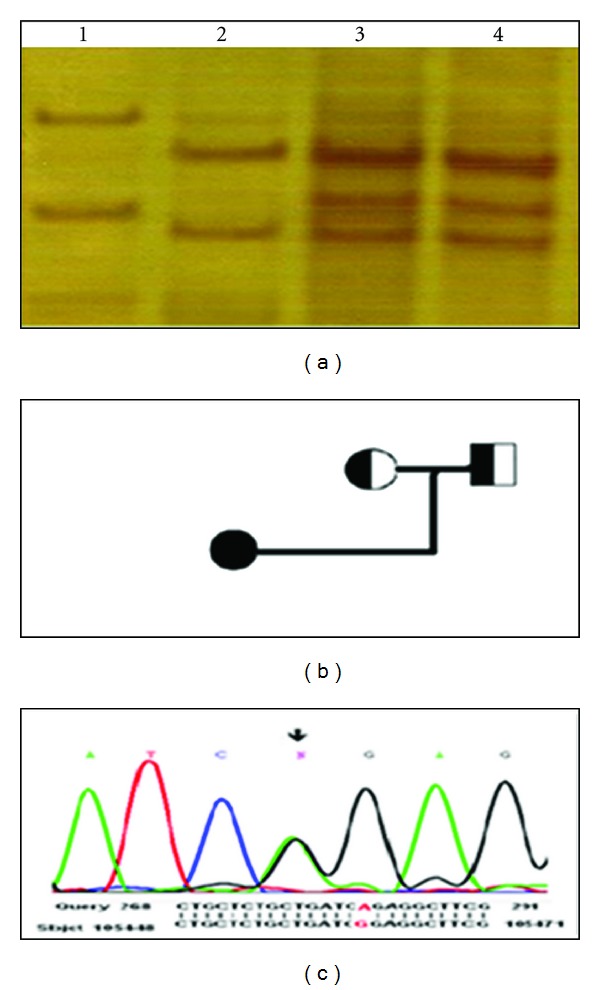
(a) SSCP analysis of exon 15 in affected family. Line1: Normal control, Line2: The patient that has homozygous G2669A mutation in exon 15, Line3: The affected s' mother, Line4: The affected s' father. (b) Pedigree of the affected family is aligned with the SSCP analysis. (c) Sequencing analysis result of affected s' mother and father.

For further analysis, SSCP and DNA sequencing of this exon were performed for the family. The data showed that the mutation is present in heterozygous form in the parents (Figure 2). The parents of the proband have had first cousin marriage.

The affected patient was characterized by SSCP as having aberrant shift in exon 15 (14th coding exon) that was not detected in normal subject and other patients and was found homozygous for this mutation in sequencing analysis result (Figure 1) SSCP of exon 15 showed altered migratory patterns in both parents of affected patient, and sequence analysis in them revealed that they carried this mutation (Figure 2).

In addition, six known single nucleotide polymorphisms were detected in this cohort by SSCP and sequencing analysis. Two of them were located in the promoter region and in exon 1 (A-35G, G11A) and others in the reading frame c.859G > T, c.1207G > T, c.1283G > C, c.2088C > T).

Full sequencing of TPO gene in four patients detected no mutation, and SSCP results and sequencing analysis results were similar in these patients.

## 4. Discussion

TPO gene mutations are the main causes of thyroid dyshormonogenesis [6–24]. In the present study, the whole gene scanning of  TPO gene by SSCP and sequencing was performed in 41 patients with permanent congenital hypothyroidism due to dyshormonogenesis. Only one mutation was detected in this group of patients. The reported mutation in this study c.2669G > A (NM_000547.5) is located in exon 15 of TPO gene and results in the hydrophobic glycin (G) to the positively charged arginine amino acid substitution in the TPO transmembrane region. This change is effected on insertion of the TPO enzyme into the plasma membrane of thyroid follicular cells, and results decrease protein activity in the patient [17]. Both parents were heterozygous for this change so the mutation transmits as autosomal recessive traits in the affected family. Seven different single nucleotide polymorphisms (SNPs) in exons 1, 7, 8, 11, and 15 of the TPO gene were detected too.

As mentioned, different mutations of TPO gene have been reported previously, commonly in exons 8, 9, and 10. The mutation of c.2669G > A (NM_000547.5) has been reported by Avbelj et al. in Slovakia, and thereafter it was not reported in other studies [17]. Mutation of exon 15 has been reported by Neves et al. in Brazil, but it was c.2630T > C mutation [32].

The patient with mentioned mutation in Slovenia had nodular goiter according to the sonographic findings at the age of 16 years [17]. In this study, the patients had not goiter according to both radiologic and clinical findings at the age of 3 years. Though it may be due to early initiation of treatment, it needs further studies in this field.

In our study, the frequency of TPO gene mutations was lower than Slovene and Portuguese population studies that had similar inclusion criteria, without doing perchlorate discharge test [17, 19].

In a population-based study in Japan, Narumi et al. have analyzed the prevalence of TPO gene mutation in fourteen CH patients with dyshormonogenesis and detected two biallelic mutations among them [33].

In this study, total iodine organification defect (TIOD) or partial iodine organification defect (PIOD) as defined by the perchlorate discharge test was not determined in studied patients. Some studies have reported that homozygous and compound heterozygous TPO gene mutations are more frequently seen among dyshormonogenic CH patients with TIOD [32, 34]. It is suggested that low frequency of TPO gene mutations in our studied population may be due to the fact that most of them were dyshormonogenic CH patients with PIOD, which should be investigated in future studies. 

In our study, number of patients and determination of permanent CH were similar to previous studies, so low sample size or transient disease cannot be the cause of low frequency of TPO gene mutations. 

It is possible that mutations in intronic sequences or in the promoter region and unexamined regulatory regions of TPO gene are the cases of thyroid dyshormonogenesis in these patients. In addition, other genetic disorders may be more effective than TPO mutations in CH patients with dyshormonogenesis including the sodium symporter (NIS) gene, the pendrin gene (PDS), the thyroid oxidase gene 2 (THOX2 or DUOX2), and thyroglobulin gene [35].

The technique used in this study, SSCP, is a cheap, simple, and suitable method with a good sensitivity. It has 70–90% sensitivity for the detection of single base substitutions [36]. In addition, small deletions and insertions in the genome could be identified by this method [37]. High prevalence of previously reported point mutations in TPO gene and high application of SSCP in detection of this type of mutations with another benefits of this method that is mentioned are reasons to choose SSCP for this study. Approximately 90% of the potential base exchanges are detectable by SSCP under optimal conditions [38]. In present study, detection of several single nucleotide polymorphisms in different regions of  TPO gene with SSCP and similarly results of direct sequence analysis in four patients showed high sensitivity of SSCP in our study. But SSCP like another mutations screening methods may have percentage of error rate. The limitations of SSCP method are high depen. So in the present study, there is probability of existing mutations that are unidentified with SSCP.

We know, long homozygous deletions in the gene are caused of autosomal recessive disorders. In this group of patients, deletion regions cannot amplify with PCR method and they are detectable with this way, but patients with long heterozygous deletions because of having one normal copy of gene are not detectable with PCR-SSCP method and sequencing analysis. So far, there is no report about long deletions related with TPO gene but the probability of existence of these type of mutationsshould be investigated in studied population in our future studies.

In conclusion, because of low prevalence of TPO gene mutation in this study, it is necessary to investigate more studies with large sample by using another screening method besides SSCP and screening of intronic and regulatory TPO gene mutations and mutation detection of other genes that had effect on thyroid dyshormonogenesis.

## Figures and Tables

**Table 1:** Primers used for PCR Amplification of thyroid peroxidase gene.

Exon	Forward Primers		Reverse Primers		Fragment size (bp)	Annealing temperature
	Position of 5^′^ end	Nucleotide sequence (5^′^→3^′^)	Position of 5^′^ end	Nucleotide sequence (5^′^→3^′^)
(1)	**−**70	GACTTCCTAGCATCTTGACG	+67	CACTTTACAAGTTCCAATGATG	220	58^°^C
(2)	−74	AGACAAGGACACAGCGGTTC	+95	CATGGCCTTGTCAGTGCTTG	225	60^°^C
(3)	−67	AAGCAACACTGTCAGTGAATC	+123	TTAACAATGGCAAGCTTCAG	275	60^°^C
(4)	−60	TT AAGTACCAAAGATACCATAGAC	+65	CACAAAGTCAAGGTGTCCTC	295	60^°^C
(5)	−101	CAAATTCAGATGCTGGAGTCAC	+73	TCCTTCATGATGGCATCTAGTC	308	61^°^C
(6)	−86	CTGAGAATGGTGTCTTATATCTG	+52	AGCATCACAGGACCCAATC	313	61^°^C
(7)	−61	GTCATCTTTCTGCTACCACG	+60	TTGACGTTTTAAATAGCACTTAG	327	55^°^C
(8)	−60	AGAGTCTTACAA AGG GTG CAC	+163	AAG TAC CTG GGA GAG AGA AGC	678	60^°^C
(9)	−29	TCA CTGAGATGCTTTTCCTAT C	+45	AAGAGTTCATGGGGACCAG	327	60^°^C
(10)	−55	GTTTCTCTAGAACTGAGCCAAG	+79	AGTCTCTCTAGCAGCAGGTTG	306	61^°^C
(11)	−51	AACAAAAGTTCAGTTCTGTGAGAG	+44	TGTGCAGAACGTGAAGGAAG	330	61^°^C
(12)	−42	CTC CAT GCA CTG TGA CCT TAC	+57	CTTTGTTTGATGAGATGCACG	308	61^°^C
(13)	−46	CTTTTCTCGTAGTTTGACTACATG	+54	CTTATATCGGAAACATTCAGATG	271	60^°^C
(14)	−69	AGAGAAGCACCTCCCAGAAC	+69	TACAAAAACTCGCAAATGGAC	270	61^°^C
(15)	−75	CAGACTCAGGCAGGACAACC	+69	ATTGCAGCCATGTCCAGAG	244	61^°^C
(16)	−61	CTACCCTCCACAGTCACGGT	+59	CCAGATCCTGTCCAACCACT	250	62^°^C
(17)	−108	TGTGAAAAGAGCTCCTGTC	+49	GTGATTTTGGGAACATGAAG	211	62^°^C
